# Psychological distress, quality of life, needs, and resources among informal caregivers in specialist palliative home care

**DOI:** 10.1007/s00520-025-10072-0

**Published:** 2025-11-17

**Authors:** Anneke Ullrich, Eva Wortberg, Carsten Bokemeyer, Karin Oechsle

**Affiliations:** 1 Palliative Care Unit, Department of Oncology, Hematology and BMT, University Medical Center Hamburg-Eppendorf, Martinistr. 52, Hamburg, 20246 Germany; 2 Palliative Care Team Aurich/Ostfriesland GmbH, Aurich, Germany

**Keywords:** Home care, Informal caregiver, Palliative care, Psychological distress, Quality of life, Needs

## Abstract

**Purpose:**

Informal caregivers (ICs) of patients with advanced incurable diseases experience psychological distress, unmet needs, and impaired health-related quality of life (HRQOL). While research on ICs in inpatient palliative care is expanding, less is known about their specific problems, needs, and resources in palliative home care settings.

**Methods:**

This cross-sectional, single-center study consecutively recruited 76 adult ICs within 72 h of initiating specialist palliative home care. ICs completed standardized questionnaires assessing psychosocial distress, anxiety, depression, importance and fulfilment of needs, HRQOL, social support, and sources of meaning. Descriptive and regression analyses were conducted to explore associations between outcomes.

**Results:**

The results revealed that 78% of ICs experienced clinically relevant psychosocial distress, with 46% experiencing severe distress. Over one-third of ICs showed signs of anxiety or depressive disorders. HRQOL scores were significantly lower than those of the general population, particularly in mental health and emotional role functioning. About one-third of IC’s needs were unmet, especially regarding emotional support (e.g., feeling hope) and treatment information. Personal relationships and financial security were key sources of meaning, and 77% reported moderate to strong social support. Higher distress, anxiety and depressive symptoms were each significantly associated with lower mental and physical HRQOL.

**Conclusion:**

ICs in palliative home care experience substantial psychological distress, impaired HRQOL, and unmet needs. To improve their well-being, it is crucial to address emotional and informational needs, enhance social support, and promote sources of meaning. Tailored interventions are essential to support ICs in the setting of palliative home care.

**Supplementary Information:**

The online version contains supplementary material available at 10.1007/s00520-025-10072-0.

## Background

Psychosocial burden, unmet needs and impaired health-related quality of life are common among informal caregivers (ICs) of patients with advanced incurable diseases [1]. Given the critical role ICs play in supporting patients receiving palliative care, along with the negative outcomes often associated with such caregiving, the goals of palliative care include to support ICs and, in turn, improve the patient’s care experience. Palliative care services are offered in both inpatient and home settings, with the latter serving as a main delivery model of palliative care [2]. However, while there is a growing body of research focusing on inpatient palliative care settings, such as palliative care wards [3–9], studies examining the specific problems, needs, and resources of ICs in palliative home care are less frequent. A study by Tanco et al. suggests that the burden on ICs may differ across palliative care settings [10], emphasizing the need for a specific perspective on palliative home care.

A Canadian study reported that 22% of ICs experienced caregiver distress during palliative home care, identifying both patient- and caregiver-related factors that increase the risk of this distress [11]. In addition, interactions between the various challenges and demands faced by ICs have been observed, such as the link between higher levels of unmet needs with poorer quality of life [12]. A key stressor for ICs in palliative home care appears to be the tension between control and loss of control [13], where personal problems, own needs and resources play a critical role. Studies also show dyadic effects between the psychosocial problems and needs of ICs and patients during palliative home care [14], indicating that supporting ICs may improve the patients’ situation as well. Additionally, research demonstrates that ICs’ distress can persist into the bereavement period following palliative home care [15]. Fortunately, early evidence indicates that standardized assessments and support interventions can have a positive impact on ICs during palliative home care [16–19], emphasizing the importance of better understanding ICs’ needs and implementing tailored support programs [20, 21]. Previous research by our study group has also investigated psychological distress—comprising psychosocial distress, anxiety and depression—along with needs and HRQOL in a cohort of ICs of cancer patients entering specialist palliative care. However, this research has been limited to the inpatient setting of palliative care wards [3–5].

The current study aimed to analyze the psychological distress, health-related quality of life (HRQOL), the importance and fulfillment of needs, as well as potential resources—including social support and sources of meaning—in ICs of patients with advanced incurable diseases, both cancer and non-cancer, receiving specialist palliative home care. Additionally, the study explored potential relationships between ICs’ psychological distress and their HRQOL, needs, and resources. The measurements used were identical to those in our previous research on ICs in palliative care wards, allowing for comparability.

## Methods

### Study design and participants

This exploratory, single-center, cross-sectional study recruited ICs of patients with advanced incurable diseases within 72 h of initiating specialist palliative home care provided by the Palliative Care Team Aurich/Ostfriesland GmbH, Germany.

The eligibility criteria were: (1) being an IC of an adult patient with an advanced incurable disease (both cancer and non-cancer) currently living at home; (2) having a significant personal relationship with the patient, such as family members and friends who provide unpaid care or support; (3) being over 18 years of age; and (4) possessing sufficient cognitive and language skills to participate in the study, as assessed by the responsible palliative care physician. Individuals who had only a legal caregiving relationship with the patient were excluded.

The procedures of this study followed the Strengthening the Reporting of Observational Studies in Epidemiology (STROBE) guidelines [22]. Within 72 h of initiating specialist palliative home care, the responsible palliative care physician consecutively invited ICs to participate. ICs completed a pseudonymized questionnaire, which was then returned to the physician in a sealed envelope, either in person or by mail. Participation in the study was limited to one IC per patient.

Written informed consent was obtained from all ICs prior to their inclusion in the study. Ethical approval.

was granted by the Ethics Committee of the Medical Association in Hamburg, Germany (No. PV 5122). The data were provided to the study group in a non-identifiable format by the responsible palliative care physician.

### Instruments

ICs completed a set of standardized, validated self-report instruments assessing psychological distress (including psychosocial distress, anxiety, and depression), HRQOL, the importance and fulfillment of needs, and resources (including social support and sources of meaning). Additionally, sociodemographic information about both ICs and patients, as well as care-related data, were collected from ICs using study-specific items. These items were similar to those employed in previous research on ICs in specialist inpatient palliative care conducted by the study group [3, 4].

#### Psychological distress

*NCCN Distress-Thermometer (DT):* The DT [23] was used to assess psychosocial distress in ICs over the past 7 days on a visual analogue scale from 0 (no distress) to 10 (extreme distress). In the validated German version [24], a score of ≥ 5 indicates clinically relevant distress, which has also been confirmed for ICs [25]. Based on our previous research [3], a cut-off of ≥ 8 was applied to identify those with “severe” distress requiring urgent intervention. An adapted problem list for ICs was also used [3, 4].

*General Anxiety Disorder 7-item Scale (GAD-7):* The GAD-7 [26] was used to measure ICs’ anxiety and consists of seven items scored on a 4-point Likert scale from 0 (not at all) to 3 (nearly every day). The sum score ranges from 0 to 21, which can be categorized into minimal (0–4), mild (5–9), moderate (10–14) and severe (15–21) symptoms of anxiety. A cut-off score of ≥ 10 indicates a possible anxiety disorder. In the present study, the German version of GAD-7 [27] demonstrated good internal consistency (Cronbach’s alpha = 0.859).

*Patient Health Questionnaire – depression module 9-item scale (PHQ-9):* The PHQ-9 [28] was used to measure ICs’ depressive symptoms and consists of nine items scored on a 4-point Likert scale from 0 (not at all) to 3 (nearly every day). The sum score ranges from 0 to 27, which can be categorized into minimal (0–4), mild (5–9), moderate (10–14) and severe (15–27) depressive symptoms. A cut-off score of ≥ 10 indicates possible depression. In the present study, the Cronbach’s alpha for the German version of PHQ-9 [29] was 0.869.

#### Health-related quality of life

*Short Form-8 Health Survey (SF-8):* The SF-8 is a validated short version of the Health Survey Form-36 [30], designed to assess various aspects of health. It consists of eight items, which generate eight sub-scales and two component scores (physical and mental), all calculated using norm-based scoring methods. Higher scores (0–100 scale) indicate better HRQOL. Age- and gender-adjusted data for adults are available for the German version [31].

#### Needs

*Family Inventory of Needs (FIN):* The FIN [32] consists of 20 items designed to assess the importance and fulfilment of needs [33]. According to the German version [34], ICs rated the FIN-Importance scale on a 5-point Likert scale, ranging from 1 (not important) to 5 (extremely important). Scores were collapsed into “important” (4 or 5) and “not important” (1, 2 or 3). The FIN-Importance total score reflects the number of needs rated as important (0–20 scale). For the FIN-Fulfilment scale, ICs rated whether a need was met (1), partly met (0.5) or unmet (0). Scores were grouped into “unmet” (0 or 0.5) and “met” (1). The FIN-Fulfilment total score represents the number of needs rated as unmet (0–20 scale).

#### Resources

*Oslo Social Support 3-item Scale (OSLO-3):* The OSLO-3 [35] was used to assess the perceived extent of social support. The total score ranges from 3 to 14 and is calculated by summarizing the raw scores of the items. Scores between 3–8 reflect “poor”, 9–11 “moderate” and 12–14 “strong support” [35].

*Sources of Meaning Profile (SOMP-R):* The SOMP-R [36] consists of 17 items designed to evaluate sources that provides individuals with a greater sense of meaning in their present life. Each item is rated on a 7-point Likert scale, ranging from 1 (not at all meaningful) to 7 (extremely meaningful). Scores of ≥ 5 are considered highly meaningful or important. A breadth score is calculated as an index of the diversity of these highly meaningful sources (0–17 scale).

### Statistical analyses

Descriptive statistics were calculated, including frequency distributions, percentages, means, and standard deviations. For calculations of total scores, missing values were replaced by the individuals’ mean value if fewer than 20% of items were missing. A one-sample *t*-test was used to assess differences in mean values. Effect sizes were determined using Cohens’s *d*, with 0.2 ≤ *d* < 0.5 considered a small effect size, 0.5 ≤ *d* < 0.8 a medium effect size, and *d* ≥ 0.8 a large effect size [37]. Multiple linear regression analyses (backwards selection procedure) were conducted to examine the relationship between continuous outcomes of psychological distress (dependent variables: psychosocial distress, anxiety, and depression scores) and HRQOL (independent variables: physical and mental component scores), needs (independent variables: importance and fulfilment scores), and resources (independent variables: social support score and sources of meaning score). Based on the rule of thumb, the sample size should be at least 10 participants per independent variable for sufficient power to detect associations in regression models [38]. This study included six independent variables. Diagnostics for potential multicollinearity were performed [39], and no issues with tolerance values or variation inflation factors (VIF) were found.

All significance tests were two-sided using a significance level of α < 0.05. The statistical analysis was performed using the SPSS software package (SPSS version 29.0.0).

## Results

### Recruitment and characteristics

During the study period (January 6th to March 10th 2023), 169 ICs were eligible; 92 declined participation (54%), and 77 ICs completed the questionnaire; however, one questionnaire had to be excluded retrospectively due to formal constraints. Thus, 76 ICs were included in the final analysis. Details of the IC selection process are provided in Figure S1 (supplemental material).

The mean age of the ICs was 61.8 years, with nearly two-thirds (63.2%) being women. In terms of the caregiving relationship, 52.6% were spouses/partners, and 36.8% were adult children. Overall, more than three-quarters of the patients (76.3%) had cancer. The characteristics of the ICs are detailed in Table 1.

**Table 1:** Characteristics of informal caregivers and patients (N = 76)

Characteristics		*n (%)*
*Informal caregivers*		
Age, years: *M (SD), Range*		61.8 (11.8); 26–83
Gender	Male	28 (36.8)
	Female	48 (63.2)
Relationship to the patient	Spouse or partner	40 (52.6)
	Adult child	28 (36.8)
	Parent	2 (2.6)
	Sibling	5 (6.6)
	Friend	1 (1.3)
Marital status	Married	64 (84.2)
	Single	6 (7.9)
	Divorced	3 (3.9)
	Widowed	3 (3.9)
Net household income per month	< 1750 €	20 (27.8)
	1750 – ≤ 3000 €	26 (36.1)
	> 3000 €	26 (36.1)
Education	Elementary school (≤ 9 years)	32 (42.1)
	Junior high school (10 years)	26 (34.2)
	High school (12–13 years)	18 (23.7)
Employment	Employment^a^	30 (39.5)
	Retirement/Pension	43 (56.6)
	Unemployed/Housewife/-man	3 (3.9)
*Patients*		
Patient age in years	31–50	3 (3.9)
	51–60	5 (6.6)
	61–70	25 (32.9)
	> 70	43 (56.6)
Patient gender	Male	44 (57.9)
	Female	32 (42.1)
Patient primary diagnosis	Cancers of the male genital organs	14 (18.4)
	Cancers of the respiratory system	11 (18.4)
	Cancers of the digestive system	12 (15.8)
	Breast cancer	9 (11.8)
	COPD	8 (10.5)
	Heart diseases	4 (5.3)
	Other cancers	4 (5.3)
	Other non-cancer diseases	4 (5.3)
	Eye, brain, and CNS cancers	3 (3.9)
	Leukemia and Lymphoma	3 (3.9)
	Oral cancers	3 (3.9)
	Dementia	2 (2.6)

*CNS* central nervous system, *COPD* chronic obstructive pulmonary disease

^a^Includes part-time employed

### Psychological distress

Scores and prevalence of psychosocial distress, anxiety and depression are presented in Table 2. Over three-quarters of ICs (78.9%) reported clinically relevant distress (cut-off ≥ 5), with nearly half (46.1%) experiencing severe distress (cut-off ≥ 8). The most common distress-causing problems included sadness (70.3%), exhaustion (70.3%), worry (69.7%), fears (64.5%), and sleep problems (58.1%), each reported by more than half of the ICs (for the full list see Figure S2 in the supplemental material). Based on screening instruments, 36.0% of ICs scored ≥ 10 on the GAD-7 and 33.3% scored ≥ 10 on the PHQ-9, indicating elevated anxiety and depressive symptomatology at a level consistent with possible disorder (i.e., not diagnostic).

**Table 2:** Distress, depressive and anxiety symptoms of informal caregivers (N = 76)

Psychological distress		n (%)
*Psychosocial distress (DT; scale: 0–10)*		
Total score: M (SD), Range		6.6 (2.4); 0–10
Clinically relevant psychosocial distress	Yes (≥ 5)	60 (78.9)
Severe psychosocial distress	Yes (≥ 8)	35 (46.1)
*Anxiety symptoms (GAD-7; scale: 0–21)*		
Total score: M (SD), Range		8.1 (5.0); 0–19
Symptomatology at a level consistent with possible disorder	Yes (≥ 10)	27 (36.0)
Severity of anxiety symptoms	None (0–4)	18 (24.0)
	Mild (5–9)	30 (40.0)
	Moderate (10–14)	18 (24.0)
	Severe (15–21)	9 (12.0)
*Depressive symptoms (PHQ-9; scale: 0–27)*		
Total score: M (SD), Range		7.2 (5.4); 0–24
Symptomatology at a level consistent with possible disorder	Yes (≥ 10)	25 (33.3)
Severity of depressive symptoms	None (0–4)	31 (41.3)
	Mild (5–9)	19 (25.3)
	Moderate (10–14)	19 (25.3)
	Severe (15–27)	6 (8.0)

*M* Mean, *SD* Standard deviation, *DT* Distress Thermometer, *GAD-7* Generalized Anxiety Disorder 7-item scale, *PHQ-9* Patient Health Questionnaire – depression module 9-item scale

### Health-related quality of life

The HRQOL (0–100 scale) of ICs was compared to an age- and gender-matched reference group from the general German population, with detailed results presented in Table 3. ICs reported lower HRQOL than the reference group, with the largest differences observed in the “mental health” domain (ICs: M = 44.1, SD = 9.5 vs. reference sample: M = 51.9, SD = 0.9; p < 0.001, large effect) and the “emotional role functioning” domain (M = 44.1 vs. M = 47.8; p < 0.001, medium effect). In the physical domains, ICs also showed significantly lower scores in the “physical role functioning” domain (M = 44.0, SD = 8.0 vs. M = 47.2, SD = 2.1; p < 0.001) and the “general health” domain (M = 43.7, SD = 6.7 vs. M = 46.0, SD = 2.0; p < 0.001), although these differences were small in effect size. None of the differences were clinically relevant (10 points or more).

**Table 3:** Health-related quality of life of informal caregivers (N = 76) compared to an age- and gender-adjusted German standard population (N = 2,552)

Domains (scales: 0–100)	Health-related quality of life (SF-8)			
	Informal caregiver *M (SD)*	Reference value^a^ *M (SD)*	Significance *p*-value^b^	Effect size *Cohen’s d*
Physical component score (PCS)	45.9 (9.4)	47.1 (3.0)	0.257	0.132
Physical functioning (PF)	44.8 (8.8)	46.5 (2.4)	0.102	0.190
Physical role functioning (RP)	44.0 (8.0)	47.2 (2.1)	** < 0.001**	0.405
Bodily pain (BP)	50.0 (10.0)	51.2 (2.0)	0.290	0.122
General health (GH)	43.7 (6.7)	46.0 (2.0)	**0.003**	0.348
Mental component score (MCS)	44.6 (10.1)	52.2 (1.2)	** < 0.001**	0.758
Vitality (VT)	47.0 (8.0)	49.8 (2.3)	**0.003**	0.352
Social functioning (SF)	46.5 (9.2)	50.0 (1.1)	**0.002**	0.379
Emotional role functioning (RE)	44.1 (7.2)	47.8 (1.3)	** < 0.001**	0.517
Mental health (MH)	44.1 (9.5)	51.9 (0.9)	** < 0.001**	0.816

*M* Mean, *SD* Standard deviation

Significant differences are marked in bold

^a^age- and gender-matched German standard population (Beierlein et al., 2012 [31]); ^b^one-sample *t*-test (two-tailed)

### Needs

 Table 4 presents the importance and fulfilment of needs, ranked by the proportion of ICs who rated each need as very or extremely important. On average, ICs rated 17.8 (SD = 2.3) out of 20 needs as important. Fourteen needs were considered important by more than 90% of ICs, including all items from the “Basic Information” (4 items: 91.9–98.7%), “Information on Treatment and Care” (4 items: 90.7–98.7%), and “Patient Comfort” (2 items: 92.1–98.6%) domains. The lowest-rated need was in the “Support” domain (“have someone concerned with my health”: 39.5%).

**Table 4:** Importance and fulfilment of informal caregivers’ needs (N = 76)

Needs (FIN)	Importance subscale	Fulfilment subscale
	Important needs^a^ *n/N (%)*	Unmet needs^b^ *n/N (%)*
*Domain 1: Basic information*		
…have information given in terms that are understandable	75/76 (98.7)	22/75 (29.3)
…be informed of changes in the patient´s condition	73/76 (96.1)	21/71 (29.6)
…have my questions answered honestly	72/76 (94.7)	33/74 (44.6)
…know specific facts concerning the patient`s prognosis	68/74 (91.9)	36/72 (50.0)
*Domain 2: Information on Treatment and Care*		
…know what symptoms the treatment or disease can cause	75/76 (98.7)	34/68 (50.0)
…know what treatment the patient is receiving	73/74 (98.6)	14/72 (19.4)
…know exactly what is being done to the patient	74/76 (97.4)	20/73 (27.4)
…know why things are done for the patient	71/75 (94.7)	23/68 (33.8)
…know the probable outcome of the patient`s illness	71/75 (94.7)	34/68 (50.0)
…be told about changes in treatment plans while they are being made	69/74 (93.2)	25/67 (37.3)
…know when to expect symptoms to occur	68/75 (90.7)	39/67 (58.2)
*Domain 3: Support*		
…have information about what to do for the patient at home	70/73 (95.9)	17/68 (25.0)
…feel there is hope	60/70 (85.7)	40/67 (59.7)
…feel accepted by the health professionals	65/76 (85.5)	11/71 (15.5)
…help with the patient`s care	57/73 (78.1)	16/68 (23.5)
…know the names of the health professionals involved in the patient`s care	58/75 (77.3)	9/71 (12.7)
…be told about people who could help with my problems	55/76 (72.4)	35/73 (47.9)
…have someone be concerned with my health	30/76 (39.5)	31/76 (40.8)
*Domain 4: Patient Comfort*		
…be assured that the best possible care is given to the patient	73/74 (98.6)	25/71 (35.2)
…feel that the professionals care about the patient	70/76 (92.1)	10/76 (13.2)
Number of important^1^ or unmet^2^ needs (scales: 0–20); M (SD), range	17.8 (2.3); 8–20	7.2 (5.7); 0–19

^a^Needs rated as “very important” or “extremely important”

^b^Needs rated as “not met” or “partly met”

In terms of unmet needs, ICs reported a mean of 7.2 (SD = 5.7) out of 20 needs as not or only partly met. Five needs were unmet by more than half of ICs, most commonly in the “Support” domain (“feel there is hope”: 59.7%), followed by items from the “Information on Treatment and Care” domain (3 items: 50.0–58.2%) and “Basic information” (1 item: 50.0%) domains.

### Resources

The mean score for social support (3–14 scale) was 10.8 (SD = 2.3, range: 6–14). Most ICs reported feeling very well or well socially supported (77.7%), with 46.1% (n = 35) indicating strong support and 31.6% (n = 24) reporting moderate support. In contrast, nearly one-quarter (22.4%, n = 17) rated their social support as insufficient, with their responses classified as poor support.

Sources of meaning are detailed in Table 5. Each source is ranked by the percentage of ICs who considered it highly meaningful/important. Personal relationships were the most frequently cited (81.6%), followed by financial security (71.1%), being of service to others (68.0%), and a relationship with nature (67.1%). Other relevant sources included meeting basic everyday needs (58.7%) and engaging in hedonistic activities (57.3%). The least frequently mentioned were interest in societal/political causes (10.5%) and religious activities (8.0%). On average, ICs identified 6.5 (SD = 3.6) out of 17 sources as highly meaningful/important, reflecting the diversity of meaningful aspects in their lives.

**Table 5:** Informal caregivers’ sources of meaning (N = 76)

Sources of meaning (SOMP-R)	Highly meaningful/important^a^
	*n (%)*
*Domain 1: Self-Transcendence*	
Engaging in personal relationships with family and/or friends	62 (81.6)
Being of service to others	51 (68.0)
Relationship with nature	51 (67.1)
Preserving human values and ideals	21 (27.6)
Taking part in religious activities	6 (8.0)
*Domain 2: Collectivism*	
Interest in human rights (humanistic concerns)	28 (36.8)
Preservation of culture and tradition	27 (36.0)
Leaving a legacy for the next generation	20 (26.7)
Interest in social and/or political causes	8 (10.5)
*Domain 3: Individualism*	
Participation in leisure activities	31 (41.3)
Being acknowledged for personal achievements	21 (27.6)
Experiencing personal growth	16 (21.3)
Taking part in creative activities	14 (18.7)
*Domain 4: Self-Preoccupation*	
Feeling financially secure	54 (71.1)
Meeting basic everyday needs	44 (58.7)
Participation in ‘hedonistic’ activities	43 (57.3)
Acquiring material possessions to enjoy the good life	14 (18.7)
Number of highly meaningful sources (scale: 0–17)^a^; M (SD), range	6.50 (3.63), 0–14

^a^Sources of meaning rated ≥ 5 (ratings: 1 “not at all meaningful” to 7 “extremely meaningful”)

### Relationship between psychological distress and quality of life, needs, and resources

The results of regression analyses are presented in Table 6. In multivariable models, higher anxiety and depressive symptoms were each associated with lower mental HRQOL (*ß* = −0.569 and −0.540; both *p* < 0.001) and physical HRQOL (*ß* = −0.412 and −0.516; both *p* < 0.001). Higher psychosocial distress was associated with lower physical HRQOL (*ß* = −0.303, *p* = 0.006), lower mental HRQOL (*ß* = −0.298, *p* = 0.010), and a greater number of important needs (*ß* = 0.238, *p* = 0.039). Other variables, such as the number of unmet needs, the number of highly important sources of meaning, and social support, were not significantly related.

**Table 6:** Relationship between psychological distress and quality of life, needs, and resources. Results of linear regression analyses (N = 76)

	B (95% CI)	S.E	ß	p
Dependent variable: Psychosocial distress (scale: 0–10)				
Physical HRQOL (0–100)	−0.071 (−0.121;−0.021)	0.025	−0.303	**0.006**
Mental HRQOL (0–100)	−0.068 (−0.119;−0.017)	0.025	−0.298	**0.010**
Number of important needs (0–20)	0.238 (0.012;0.463)	0.113	0.238	**0.039**
Number of highly important sources of meaning (0–17)	−0.130 (−0.271;0.011)	0.071	−0.202	0.070
Constant	9.553 (4.086;15.021)			
R^2^: 0.400				
Dependent variable: Anxiety symptoms (GAD-7, scale: 0–21)				
Physical HRQOL (0–100)	−0.205 (−0.287;−0.123)	0.041	−0.412	**< 0.001**
Mental HRQOL (0–100)	−0.275 (−0.354;−0.195)	0.040	−0.569	**< 0.001**
Constant	29.696 (25.184;34.208)			
R^2^: 0.613				
Dependent variable: Depressive symptoms (PHQ-9, scale: 0–27)				
Physical HRQOL (0–100)	−0.285 (−0.265;−0.205)	0.040	−0.516	**< 0.001**
Mental HRQOL (0–100)	−0.289 (−0.366;−0.211)	0.039	−0.540	** < 0.001**
Constant	33.286 (28.893;37.680)			
R^2^: 0.701				

*B* Unstandardized Beta Coefficient, *CI* 95% Confidence Interval, *S.E*. Standardized Error for Beta, *ß* Standardized Beta Coefficient, *p-value* *p*, probability of type I error, *HRQOL* health-related quality of life

Independent variables initially included in the regression models (first step): Physical HRQOL (scale: 0–100), Mental HRQOL (scale: 0–100), Number of important needs (scale: 0–20), Number of unmet needs (scale: 0–20), Number of highly meaningful/important sources of meaning (scale: 0–17), and Social support (scale: 3–14)

Significant differences are marked in bold

## Discussion

The findings highlight significant psychosocial challenges faced by ICs in specialist palliative home care, including high levels of psychosocial distress, negatively affected HRQOL, and unmet needs, alongside the importance of social support and sources of meaning. These results point to the value of developing targeted interventions to support ICs in palliative home care settings.

Our study observed that 79% of ICs experienced clinically relevant psychosocial distress, with 46% reporting severe distress. Furthermore, 36% of ICs exhibited anxiety and 33% depressive symptomatology at a level consistent with possible disorder requiring intervention. These estimates are lower compared to a previous study of our study group conducted in the setting of inpatient palliative care using equivalent data sources and measurements (clinically relevant psychosocial distress: 95%, possible anxiety disorder: 47%, possible depressive disorder: 39% [3]). However, they are similar to those found in a study with ICs of cancer patients at the beginning of palliative home care, where 32% of ICs represented with high anxiety levels and 29% with high depression levels (measured by the Hospital Anxiety and Depression Scale) [40].

ICs in our study also reported significantly lower HRQOL scores compared to the general population, particularly in the domains of mental health and emotional role functioning. Although physical HRQOL was also lower, these differences were smaller, suggesting that mental health challenges may be particularly pronounced. These findings are consistent with a study that used the same HRQOL measurement (SF-8) in ICs in palliative home care [40].

Additionally, ICs reported that about one-third of their needs were unmet, particularly in the areas of emotional needs, such as feeling hope, and information on treatment and care. These findings align with previous research in the inpatient palliative care setting conducted by our study group [5], indicating that such unmet needs persist across settings. In comparison with another study in the palliative home care setting [41], roughly half of ICs in both studies reported unmet needs related to understanding the patient’s illness trajectory and anticipating the future. Notably, a higher proportion in our study reported insufficient support for their own health (40% vs. 23%). Despite differences in data collection methods — self-report versus structured nurse-led assessments—these results emphasize the need to address informational, practical, and psychosocial support for ICs in palliative care.

Social support and sources of meaning were identified as important resources for ICs. Personal relationships, financial security, and being of service to others were key sources of meaning, suggesting that enhancing ICs' sense of meaning could improve their well-being. Furthermore, ICs demonstrated diversity in the sources from which they derive meaning. It has been suggested that a broader range of important sources might be protective, as the impact of compromised meaning in one domain can be mitigated by others [42]. While most ICs reported moderate to strong social support, 22% felt their support was insufficient, indicting gaps in ICs’ resources.

Multivariable regression analyses indicated that higher psychological distress was associated with lower physical and mental HRQOL and with needs rated as important. Among HRQOL dimensions, lower mental HRQOL showed the strongest association with higher anxiety and depressive symptoms, consistent with previous studies linking ICs’ depression to mental aspects of quality of life in palliative home care settings [43]. In contrast, unmet needs, sources of meaning, and social support were not significantly associated with psychological distress. These findings suggest the value of jointly assessing both burden and well-being, as this may help identify key stressors and resources to inform adequate interventions for ICs. However, due to the cross-sectional design, these associations should not be interpreted as causal.

At the same time, the interpretation of our results must consider potential selection bias. Of 169 eligible ICs, 76 were analyzed, corresponding to a refusal rate of 54%. The comprehensive questionnaire set and the requirement to participate within 72 h of initiating specialist palliative home care may have discouraged some ICs from enrolling. Without descriptive data on nonparticipants, it remains unclear whether caregiver burden was under- or overestimated: highly distressed ICs may have declined due to time or emotional constraints, whereas those with greater needs may have been more motivated to engage.

Early assessment was deliberately chosen to capture the initial caregiving situation and to inform priorities for specialist palliative home care teams at the onset of care. The questionnaire set – previously applied in our inpatient palliative care research [3, 4, 6] – ensured methodological consistency and cross-setting comparability but may also have contributed to the high refusal rate. To reduce such bias, streamlined assessments with optional modules, flexible administration modes (paper, telephone, or electronic), and minimal data collection on nonresponders, along with multicenter recruitment and reminder procedures, should be considered to enhance representativeness.

### Limitations

This study has several strengths, including the use of validated instruments to assess both burdens and resources of ICs in a previously under-researched setting, consecutive recruitmen, and insights at a critical time point that enable identification of areas for specialist palliative home care support. At the same time, several limitations should be acknowledged. The cross-sectional design precludes causal interpretations of associations among psychological distress, HRQOL, needs, and resources. Recruitment from a single specialist palliative home care team, with a predominantly rural and female sample, limits generalizability to other regions or service models. Although participation was consecutive, the high refusal rate and lack of data on nonparticipants introduce selection bias. Reliance on self-report measures provides valuable insight into ICs’ lived experience but does not allow diagnostic confirmation of anxiety and depression. Finally, the modest sample size and multiple statistical tests may limit statistical power and precision; however, this study was exploratory in nature.

### Clinical implications

Optimal home care for patients relies on providing sufficient support to ICs, enabling them to sustain their role [44]. However, psychosocial support from trained specialists is often not systematically implemented for ICs in palliative home care. While psychosocial issues and unmet needs are high in both specialist inpatient palliative care and palliative home care, ICs receive varying levels of psychosocial support in each setting. Our findings suggest several key implications for advancing a comprehensive care and support system for ICs. The high levels of psychosocial distress and mental health symptoms reported by ICs highlight the need for routine screening and early intervention in palliative home care. Psychological counseling, respite care, peer networks, and regular assessments of ICs' needs could be crucial in preventing the mental health consequences of caregiving. Additionally, addressing unmet needs and strengthening ICs' social networks and sense of meaning through targeted support programs could further improve their well-being.

### Research implications

This study contributes to the body of research on ICs in palliative home care but highlights the need for further investigation into effective interventions, especially given that many tested interventions are still in the early stages of development [20]. Longitudinal research examining changes in ICs’ well-being over time and the role of sociodemographic and care-related factors could help tailor interventions to diverse populations. To address these gaps, our study group is conducting a large, multicenter, prospective, longitudinal study that examines the course of psychological distress, needs and predictive factors among ICs in both palliative inpatient and home care settings, starting from the initiation of specialist care. Additionally, our study focused on the perspective of ICs. However, it is crucial to deepen our understanding of how patients and their ICs' responses to the stress and demands of an advanced incurable disease are interrelated during the critical period of palliative home care, as they are not isolated individuals.

## Conclusion

ICs in specialist palliative home care reported considerable psychological distress, reduced HRQOL —particularly in mental health domains — and multiple unmet informational and emotional needs. These findings, from a single-center cross-sectional sample of ICs, underscore the need for routine screening and targeted support while acknowledging limited generalizability to all palliative home care caregivers. By highlighting key areas of psychological distress and unmet needs, this study provides evidence to inform clinical practice and future research. Low-burden, pragmatic assessments and strategies to enhance participation are needed to reduce selection bias and clarify the scope of needs across more diverse caregiving populations in specialist palliative home care.

## Supplementary Information

Below is the link to the electronic supplementary material.

ESM 1 (PDF 423 KB)

ESM 2 (PDF 595 KB)

## Data Availability

The dataset supporting the conclusions of this article is available upon reasonable request from the corresponding author (AU) and with permission of the data protection officer of the University Medical Center Hamburg-Eppendorf, Hamburg, Germany.
