# Supplementary material for: Psychological distress, quality of life, needs, and resources among informal caregivers in specialist palliative home care
Source: Support Care Cancer. 2025 Nov 17;33(12):1077. doi: 10.1007/s00520-025-10072-0 (PMC12628468; doi:10.1007/s00520-025-10072-0)

Supplementary material

Article title: Psychological distress, quality of life, needs, and resources among informal caregivers in specialist palliative home care

Journal: Supportive Care in Cancer

Authors: Anneke Ullrich (a.ullrich@uke.de), Eva Wortberg, Carsten Bokemeyer, Karin Oechsle

Figure S2: Full list of distress-causing problems (N=76)

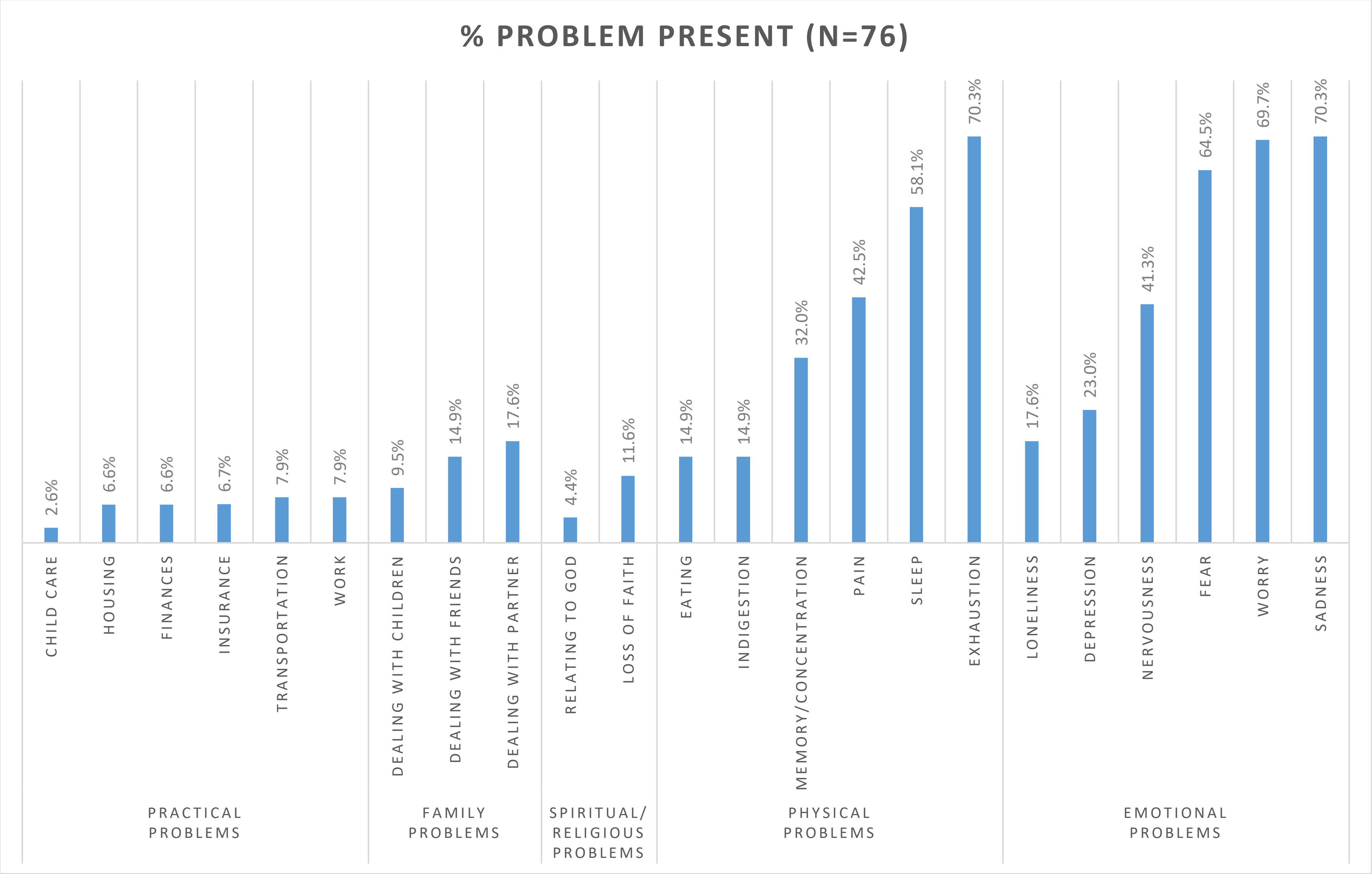

Supplement: Supplementary file 2 — (PDF 595 KB) [file 520_2025_10072_MOESM2_ESM.pdf]
